# The causality from solar irradiation to ocean heat content detected via multi-scale Liang–Kleeman information flow

**DOI:** 10.1038/s41598-020-74331-2

**Published:** 2020-10-13

**Authors:** Gang Wang, Chang Zhao, Min Zhang, Yuanling Zhang, Min Lin, Fangli Qiao

**Affiliations:** 1 The First Institute of Oceanography, State Oceanic Administration, Ministry of Natural Resources, 6 Xian-Xia-Ling Road, Qingdao, 266061 China; 2 Laboratory for Regional Oceanography and Numerical Modeling, Pilot National Laboratory for Marine Science and Technology, Qingdao, 266237 China; 3 Key Laboratory of Marine Science and Numerical Modeling (MASNUM), Ministry of Natural Resources, Qingdao, 266061 China; 4 Center for Ocean Big Data Research and Applications, National Engineering Laboratory for Integrated Aero-Space-Ground-Ocean Big Data Application Technology, The First Institute of Oceanography, MNR, Qingdao, 266061 China; 5 School of Mathematical Sciences, Ocean University of China, Qingdao, 266071 China

**Keywords:** Physical oceanography, Information technology

## Abstract

Solar irradiation is the primary driving force for the Earth’s climate system. However, we are still short of powerful tools to study the variability of the Earth’s climate due to the solar activity. Here we apply the Liang–Kleeman information flow to quantify the causality from Total Solar Irradiance (TSI) to the global ocean heat content anomaly (OHCA). It reveals that the information flow from TSI to OHCA varies in both time and space. We adapt the method into a multi-scale version which describes the variation of information flow on different timescales. In different ocean basins, the significant information flow from TSI to OHCA varies on different timescales, which could be several decades, much longer than the timescale of the correlation revealed by wavelet coherence. Then we calculate the information flow from TSI to the first three expansion coefficients of the OHCA Empirical Orthogonal Functions. The results indicate that TSI is a part cause of the El Niño-Southern Oscillation (ENSO), especially in the 1970s. In the recent 40 years, the contribution of TSI to the variation of the OHCA becomes less significant probably due to the increasing influence of human activity on the climate system.

## Introduction

Solar activity varies on timescales ranging from minutes to millennia, including such decadal or multi-decadal signals as 11-year sunspot cycle and 22-year Hale solar magnetic cycle^1^. So does the solar irradiation, one of the fundamental solar activities. Scientists believe that the variability of the total solar irradiance (TSI, also known as ‘solar constant’) affects the Earth’s climate on various timescales^2–4^.

The length of the TSI cycle is a possible indicator of solar activity associated with climate^5^. Sea temperature varies in-phase with the envelope of the 11-year solar cycle^6–8^. The decadal (8 to 15 years) and inter-decadal variability of upper ocean diabatic heat storage significantly correlated with the changing surface solar irradiative forcing^9^. The 11-year TSI cycle could force the Pacific Decadal Variability^10^. The 22-year TSI cycle prevails in the global marine temperature changes^11^. Most of the previous literature gave the correlation of solar activity and the Earth’s climate in the average temperature of global or basin scale.

However, neither the sun-climate/weather model^12,13^ nor the amplifying mechanism^14^ can explain the negative anomalies of SST in the equatorial Pacific during high solar activity years^15^. White et al.^16^ did not think that the anomalous global tropical diabatic heat storage tendency could be driven directly by the 11-year forcing of Sun’s surface irradiative, even though the global quasi-decadal signal is phase-locked to it. So far, there is no convincing explanation on how TSI acts on the Earth’s climate on different timescales, and how the climate system reacts to the changing of this forcing. There must be some unknown mechanisms to amplify the effect of the variations of solar activity on large-scale Earth’s climate^17^.

Previous researches have confirmed that ocean heat content (OHC) plays a critical role in the Earth’s heat balance in climatological annual cycle^18^ and on inter-annual-to-decadal timescales^19,20^. White et al*.*^8^ suggested that OHCA in upper ocean might also be a robust parameter to characterize the response of ocean to TSI. In some local areas, OHCA does present an obvious oscillation of 11-year period^21^.

In either natural science field or social science field, causality is always one of the most concerned issues between dynamical processes. Various methods have been introduced to study the causality between time series, for instance, lead-lag correlation, Granger Causality analysis, and information flow method. The Pearson correlation has been used to investigate the connection between solar activity and Earth’s climate. It is still widely used to explain the causality between two time series in such fields as geophysics. It should be noted that correlation does not imply causality. Besides, lead-lag correlation cannot distinguish cause and effect for periodic time series. The Granger Causality analysis, however, gives only qualitative causality relation between two time series. To measure the causality from TSI to the Earth’s climate a powerful tool suitable for a complex dynamical system is expected. Information flow used to be a terminology appeared frequently in the literature but was not rigorous. Liang and Kleeman marked an effort in putting the notion on a solid physical ground^22^. Since information flow implies causality, Liang then made a milestone in causality analysis by introducing information flow method to reveal the causal relationship between time series^23,24^. In this work, information flow specifically refers to the Liang–Kleeman information flow.

In the following, we will study the causal influence of TSI on OHCA using the information flow and its multi-scale version.

Ocean heat content is the heat contained in the ocean waters. That is

1 $$\text{Q}=\iiint {\rho C}_{p}T\text{d}x\text{d}y\text{d}z,$$

where $\rho$ is the seawater density, ${C}_{p}$ is the specific heat of seawater at constant pressure, and *T* is the temperature of the water particle in d*x*d*y*d*z*. The ocean heat content anomaly (OHCA) dataset used in this work is the product from the National Oceanographic Data Center (NODC) of the US. It gives both global spatial–temporal OHCA fields and OHCA time series of global and ocean basins from WOA09^19^. Its time ranges from 1955 to present with seasonal resolution, with the horizontal resolution of 1° by 1°.

The TSI of a few hundred years has been reconstructed based on solar activities on various timescales^25,26^. Data spanning from 1882 to the end of 2017 provides the monthly averaged climate record of TSI with associated uncertainties. To match the time resolution of OHCA data, the monthly TSI is averaged into seasonal.

## Results and discussions

### Causality from TSI to the global OHCA

**Figure 1:**
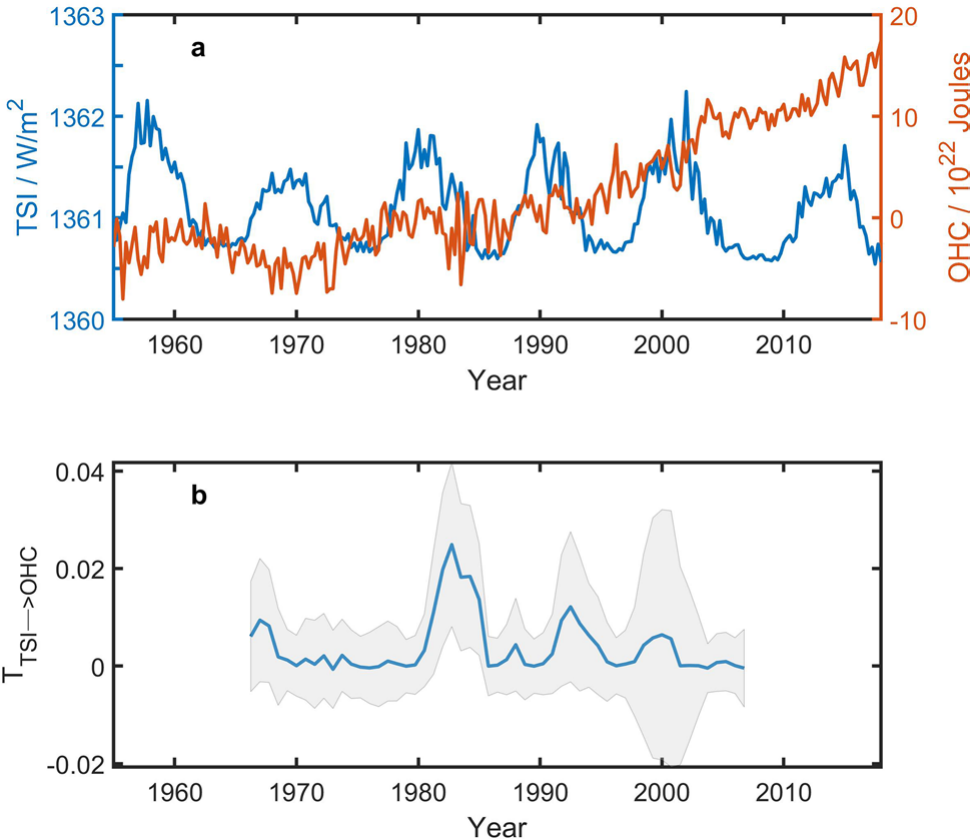
Information flow from TSI to OHCA time series. (**a**) TSI (blue line) and the global OHCA in upper 700 m (red line). (**b**) Information flow from TSI to the global mean OHCA. The size of the sliding window is 22 years (88 points), and the gray shadow covers a 68% confidence interval. The figure was created by using Matlab version R2016b.

Figure 1a shows the seasonal TSI and the global OHCA time series from 1955 to 2017 (63 years, 252 points). During this period the information flow from TSI to OHCA is 0.0051. Bootstrap test gives a confidence level of 52%. It is to say that the causality from TIS to the global mean OHCA is not significant on multi-decadal timescale. Sliding window approach (Fig. 1b) with the window size of 22 years shows that in most of 22-year period, the causality is not significant even at a low confidence level of 68% estimated by the Monte Carlo test (for variables of a normal distribution, a 68% confidence interval covers variables from one standard deviation below the mean value to one standard deviation above the mean value).

**Figure 2:**
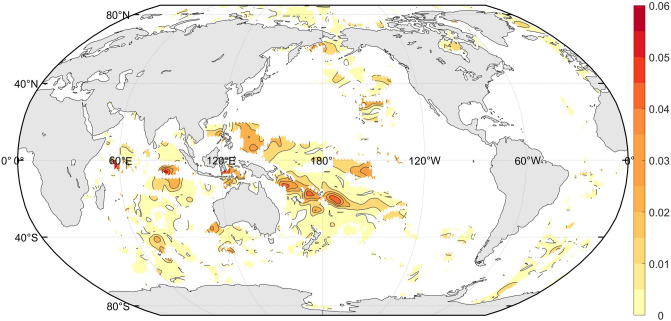
Global information flow from TSI to the gridded global OHCA in upper 700 m during the period of 1955 to 2017. Grids at which the confidence level is less than 68% are shaded blank. The map was created by using the m-map toolbox (https://www.eoas.ubc.ca/~rich/map.html) for Matlab (version R2016b).

The solar irradiation is one of the main external forcing of the Earth’s climate on decadal and longer timescales. However, as Wang et al*.*^21^ discussed that the decadal signal in OHCA is not spatially homogeneous. Through advective processes, atmosphere and oceans transport and redistribute the incoming solar radiation energy primarily in upper ocean. Therefore, the information flow from TSI to OHCA should also present local patterns. Figure 2 gives the information flow from TSI to the gridded global OHCA in upper 700 m during the period of 1955 to 2017. The information flow is significant (at a 68% confidence level) at the following areas: the South Indian Ocean, the western tropical Pacific, and the east of Australia. These areas are around the ocean conveyor belt, the well-known main surface path of the ocean current. It implies that the response of OHCA to TSI is influenced by the ocean circulation in the Earth’s climate system.

**Figure 3:**
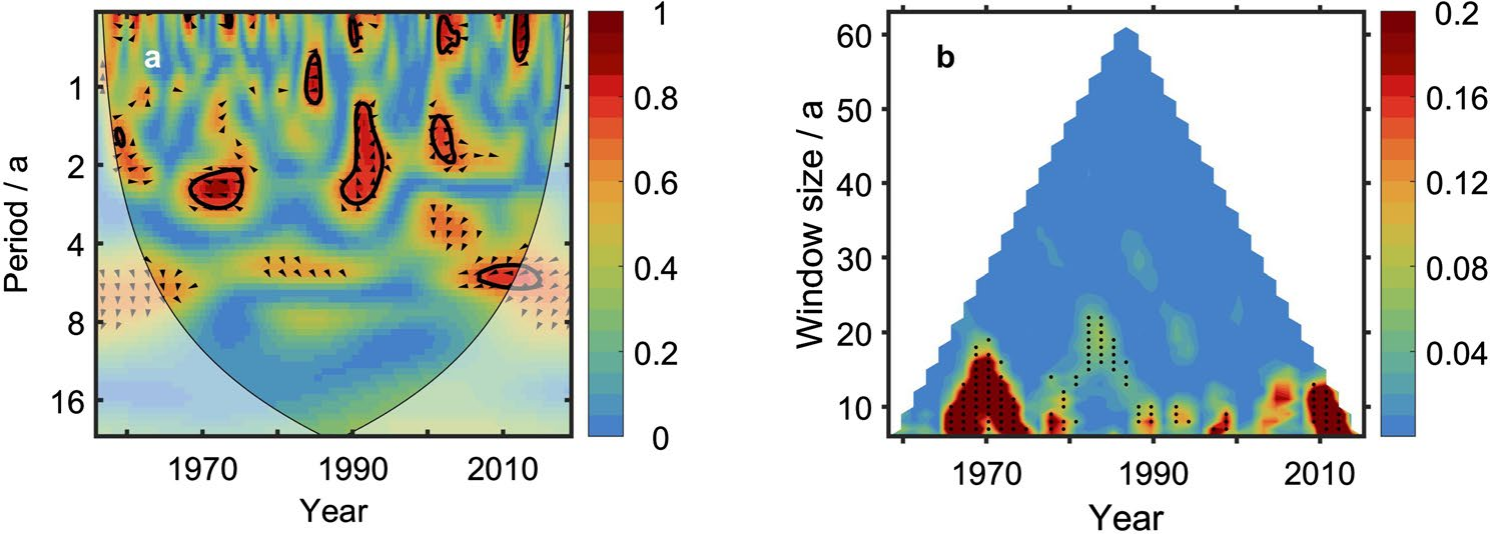
Wavelet coherence between TSI and global OHCA time series and the information flow from TSI to the global OHCA time series. (**a**) Wavelet coherence between TSI and global OHCA time series shows correlation between TSI and OHCA on inter-annual timescales. The figure was created by using the wavelet toolbox included in Matlab version R2016b. (**b**) Multi-scale information flow from TSI to global OHCA reveals causality from TSI to OHCA on decadal or longer timescales. The black dots denote that the information flow is significant at a 90% confidence level. The figure was created by using Matlab version R2016b.

Figure 3a gives the wavelet coherence between TSI and OHCA time series. Significant correlation is found only in periods shorter than 3 years. The multi-scale information flow (Fig. 3b), however, indicates that the TSI is still a cause of OHCA on longer timescales from decadal to 25 years. On these scales, the information flow from TSI to OHCA is significant at a 90% confidence level. On timescale longer than 25 years, no significant (at a 90% confidence level) information flow from TSI to OHCA is found. Around the two super El Niño events (1982–1983 and 1997–1998), the information flow from TSI to OHCA is small and not significant (at a 90% confidence level). The strong inter-annual oscillation signal of El Niño-Southern Oscillation (ENSO) overwhelms the rather slight decadal signals.

### Information flow from TSI to OHCA in the ocean basins

OHCA product provided by the NODC includes the time series of the five oceans, and their sections in each hemisphere. As is shown in Fig. 2 that the information flow from TSI to OHCA is not significant (at a 68% confidence level) in all the oceans. The dramatic increase of the global OHCA (top 2000 m) justified that the upper 700 m OHCA in the North Atlantic is an energy source to the Northern Hemisphere temperature changes^27^. Figure 2 also reveals that OHCA in the Indian Ocean presents clear response to TSI. In the following we will discuss the information flow from TSI to OHCA in the Atlantic and the Indian Ocean.

**Figure 4:**
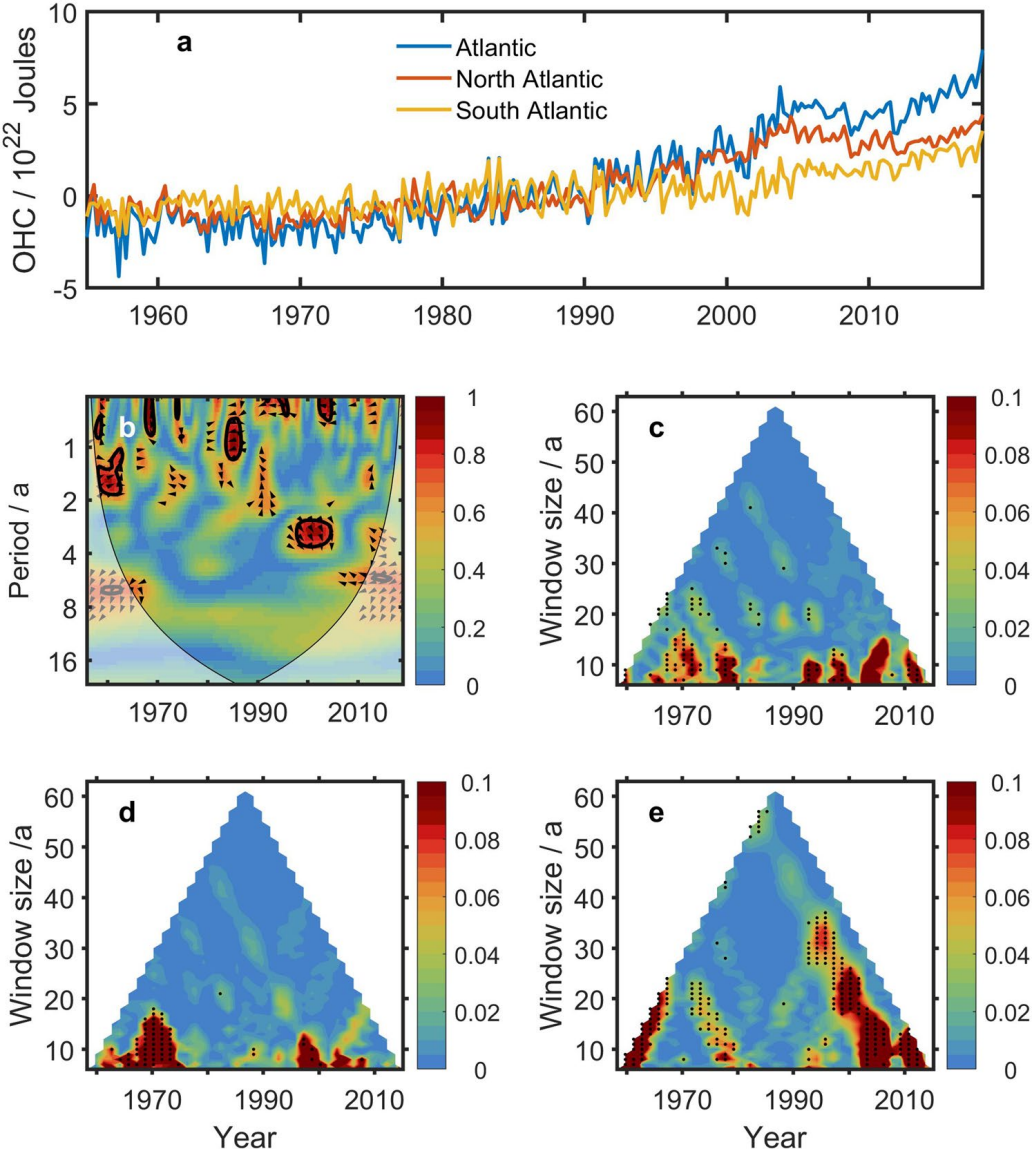
The information from TSI to OHCA in the Atlantic. (**a**) OHCA in the Atlantic, North Atlantic and South Atlantic, respectively. (**b**) Wavelet coherence between TSI and OHCA in the Atlantic. (**c**–**e**) Multi-scale information flow from TSI to OHCA in the Atlantic (**c**), North Atlantic (**d**) and South Atlantic (**e**), respectively. The black dots denote that the information flow is significant at a 90% confidence level. The figures was created by using Matlab version R2016b.

Figure 4a gives the OHCA in the whole Atlantic, North Atlantic and South Atlantic, respectively. Wavelet coherence between TSI and OHCA in the Atlantic (Fig. 4b) is significant (at a 95% confidence level) in inter-annual periods. The information flow method, however, reveals significant (at a 90% confidence level) causality from TSI to OHCA on 10–25-year timescales (Fig. 4c). It is consistent with that from TSI to the global OHCA (Fig. 3b). On the other hand, the information flows from TSI to the respective North and South Atlantic represent quite different features on timescales. For the North Atlantic, the information flow is significant on timescales less than 20 years (Fig. 4d); For the South Atlantic, however, it is significant even on timescale of 40 years around 2000 (Fig. 4e).

**Figure 5:**
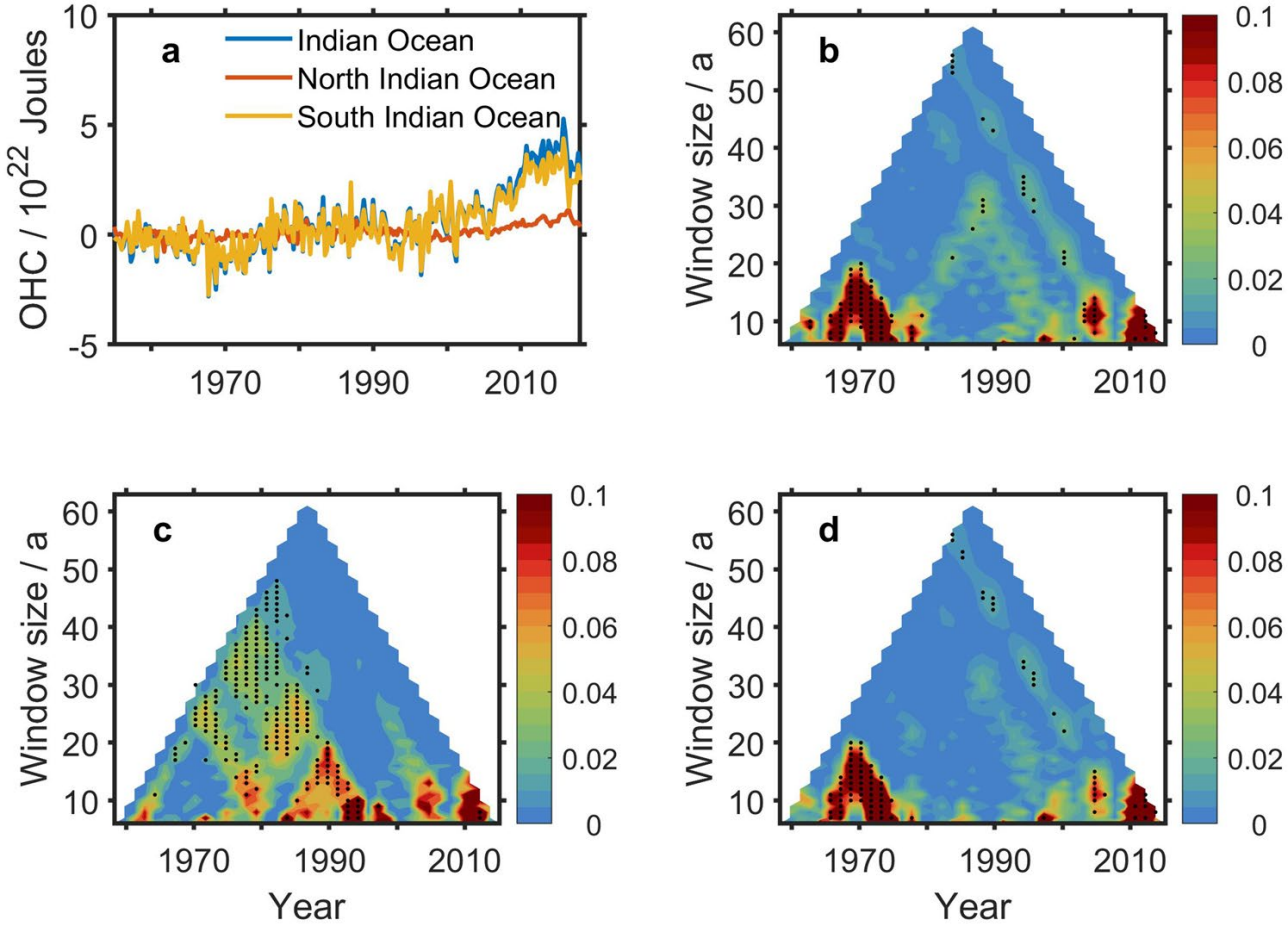
Information flow from TSI to OHCA in the Indian Ocean. (**a**) OHCA in the Indian Ocean, North Indian Ocean and South Indian Ocean, respectively. (**b**–**d**) Multi-scale information flow from TSI to OHCA in the Indian Ocean (**b**), North Indian Ocean (**c**), and South Indian Ocean (**d**), respectively. The black dots denote that the information flow is significant at a 90% confidence level. The figure was created by using Matlab version R2016b.

The main body of the Indian Ocean lies in the Southern Hemisphere. Correspondingly, the variation of OHCA in the Indian Ocean is greater than that in the Southern Hemisphere (Fig. 5a). As a result, the pattern of information flow from TSI to OHCA in the whole Indian Ocean (Fig. 5b) is similar to that in the South Indian Ocean (Fig. 5d). They are also time-dependent and timescale-dependent. On timescale longer than 40 years, for timeseries whose centers are in 1970-1990 there is significant (at a 90% confidence level) information flow from TSI to OHCA in the North Indian Ocean (Fig. 5c).

### Timescale of the information flow

**Figure 6:**
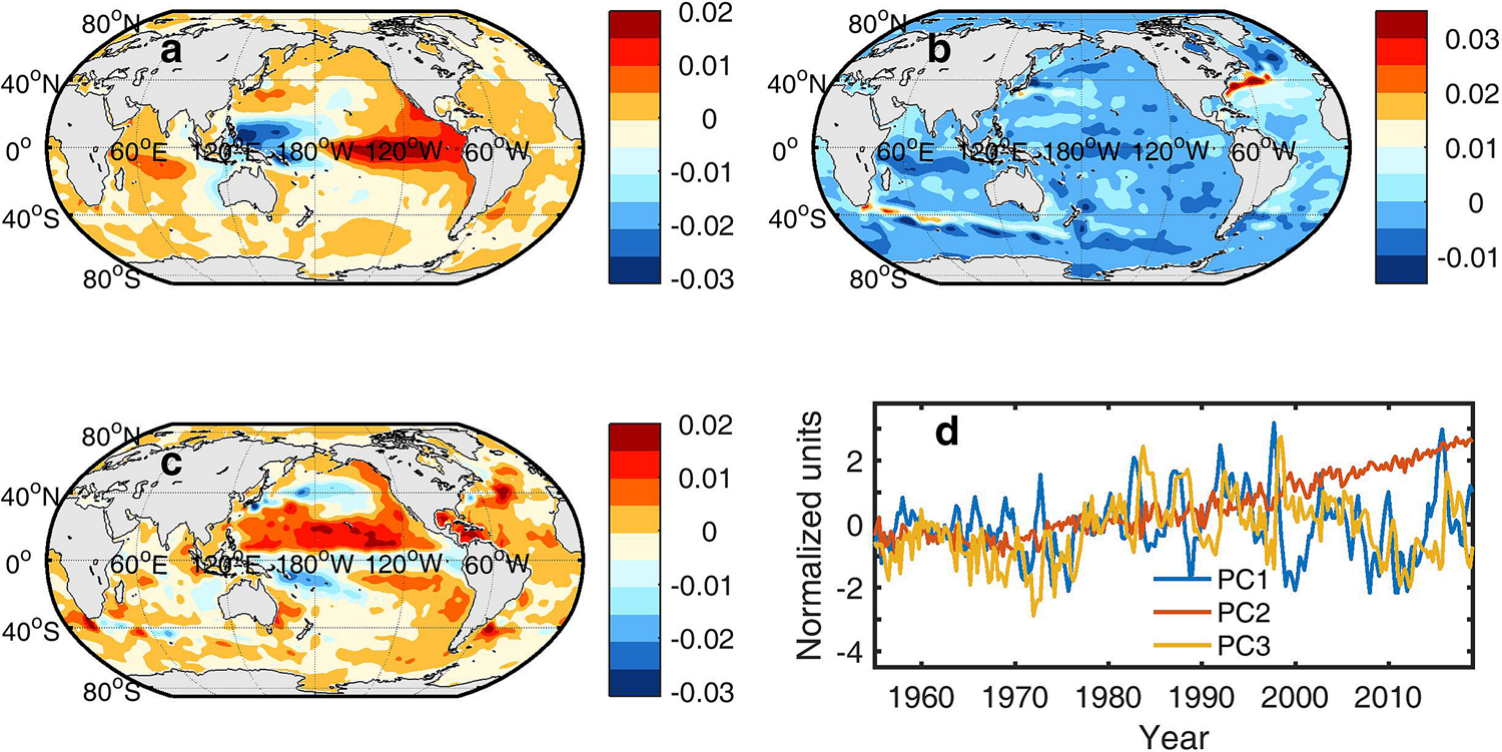
The three leading EOFs of the global OHCA and their principle components. (**a**–**c**) The three leading EOFs of the global upper ocean (top 700 m) heat content, respectively. The maps were created by using the m-map toolbox (https://www.eoas.ubc.ca/~rich/map.html) for Matlab (version R2016b). (**d**) The PCs (normalized by their respective standard deviations) of the three leading EOFs.

The previous discussion shows that the information flow from TSI to OHCA varies in space, time, as well as timescale. On the other hand, the oscillation of OHCA is influenced by various oceanic or atmospheric physical processes. We use the Rotated Empirical Orthogonal Functions (REOF) method to decompose the global OHCA into several modes (EOFs). Figure 6a–c give the three leading EOFs. EOF1 (Fig. 6a) is an El Niño-like mode. It explains 29.6% variation of OHCA. EOF2 (Fig. 6b) is a global warming mode. It explains 25.6% variation of OHCA. In this mode there are three obviously positive anomaly areas: Gulf Stream and its extension regions, Kuroshio and its extension regions, and the north boundary of the Antarctic Circumpolar Current. In these areas, Wu et al*.* detected accelerated warming. They suspected that it is possibility due to the increasing contribution of greenhouse-gas forcing to the Earth’s climate^28^^.^ EOF3 (Fig. 6c) dominates by positive OHCA especially in the North Pacific. It explains 9.4% of the OHCA variation. Figure 6d gives the expansion coefficients (also known as principle components, PCs) of the three EOFs.

**Figure 7:**
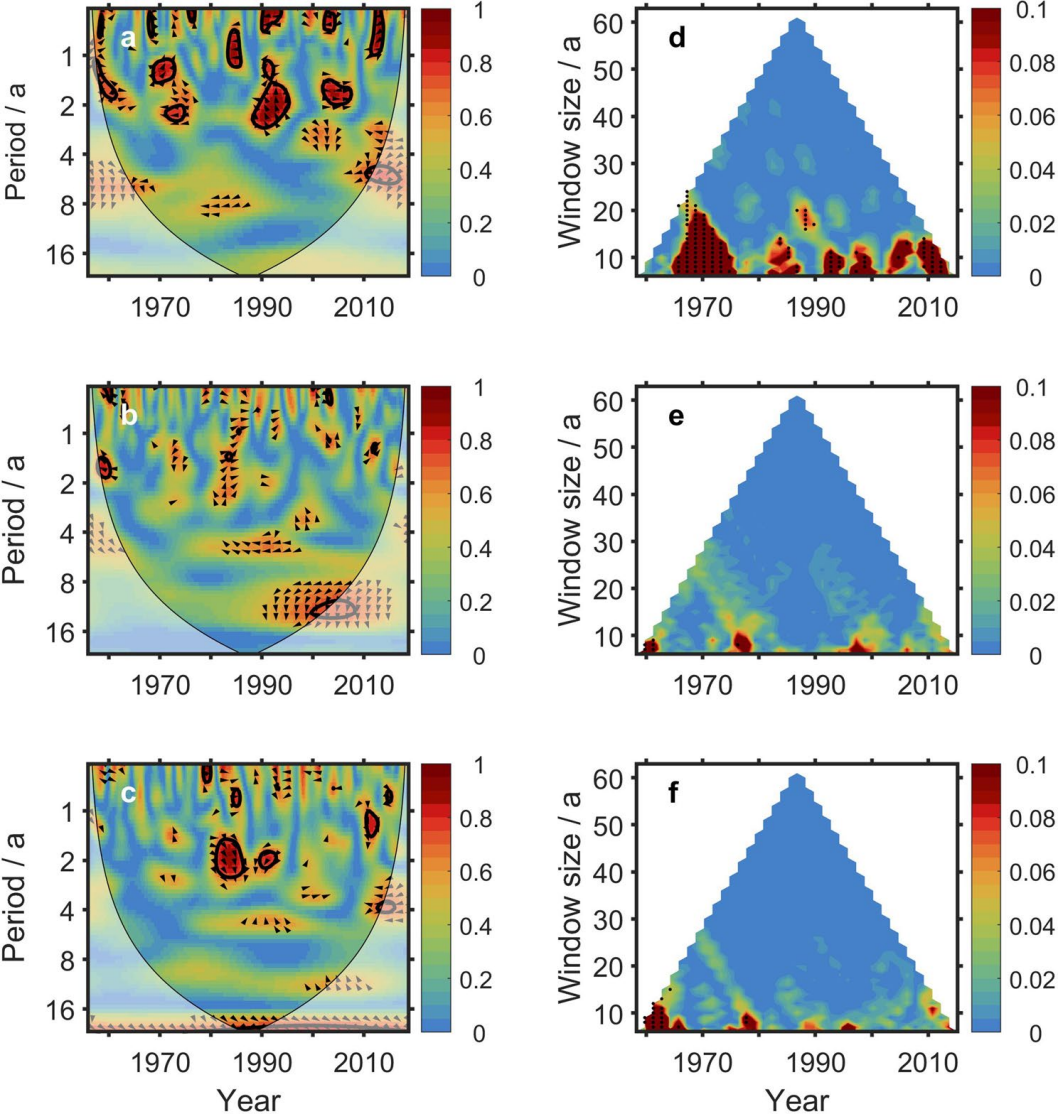
Information flow from TSI to global mean OHCA on different time scales. (**a**–**c**) Spectra of wavelet coherence between TSI and PC1 (**a**), PC2 (**b**) and PC3 (**c**) of the EOFs of global OHCA in upper 700 m. (**d**–**f**) Multi-scale information flow from TSI to PC1 (**d**), PC2 (**e**) and PC3 (**f**), respectively. The black dots denote that the information flow is significant at a 90% confidence level. The figure was created by using Matlab version R2016b.

Wavelet coherence analysis shows that TSI correlates mainly with PC1 (Fig 7a) on inter-annual timescales, and nearly no significant correlation to PC2 and PC3 (Fig. 7b–c).

White and Liu^29^ suspected that the 11-year solar activity could affect the El Niño process. Vakulenko and Sonechkin^30^ confirmed a significant 11-year solar cycle in the dynamics of the ENSO processes. Cheng et al*.* depicted a path that TSI acting on OHCA in equatorial Indian Ocean^31^. They discussed that the anomalous subsidence over the equatorial Indian Ocean provides surface downwelling solar irradiation and lighter winds to drive an OHCA increase. The lead/lag difference in net surface heat flux and OHCA tendency change reinforced the reduced Indonesian Throughflow during and after El Nino. It makes a connection between TSI and PC1. The multi-scale information flow (Fig. 7d) shows that the causality from TSI to PC1 is significant (at a 90% confidence level) in 1970s. Since 1980s, with the enhancing of El Niño signals, information flow from TSI to PC1 is overwhelmed. On timescales greater than 10 years, the causality from TSI to PC1 is not significant any more at the 0.1 level (one tailed test).

Beer et al*.*^32^ estimated that about 40% of the global warming during 1850 and 1990 was contributed by TSI. Further estimation gave the similar result: 45–50% of the 1900–2000 global warming and 25–35% of the 1980–2000 are due to TSI^33^. However, there is no significant (at a 90% confidence level) causal influence from TSI to PC2 (Fig. 7e). It is no surprise. Stips et al*.*^34^ found that the causality contribution from solar irradiation to long term trend of the global mean surface air temperature anomalies is not significant. In their work, there is a sharp substantial increase in global information flow from radiative CO_2_ forcing to the global mean temperature anomalies since the late 1970s. Jones and Moberg^35^ calculated the temperature change explained by the linear trend over several periods. In four different datasets, the trends of temperature over the period of 1945 to 1976 are negative (from − 0.039 to − 0.015), while that over the period of 1977 to 2001 are positive (from 0.137 to 0.253). The world has warmed greatly since 1976. With the accelerating of global warming due to human activities, the greenhouse contributes higher portion to the global warming than solar activity does. Simon et al*.*^36^ quantitatively estimated the Earth’s global-mean temperature increase occurring from 1910 to 1940 and from 1970 to the late 1990s. They reported that the warming from 1946 to 1996 attribute largely to the anthropogenic components. von Scheckmann et al*.* also discussed that the Earth’s energy imbalance on decadal timescales has become increasingly dominated by the anthropogenic influences^37^. The standard deviation in monthly Earth’s energy imbalance anomalies is approximately 0.6 W/m^2^, and that associated with solar forcing over the 11-year solar cycle accounts for approximately one sixth of it. These above works indicate that the 1970s is a turning point that the causal influence of solar irradiance to the global OHCA weakens.

Information flow from TSI to PC3 is significant (at a 90% confidence level) only before 1970s (Fig. 7f).

## Conclusions

There have been long-term debates on the role of solar activity in the climate change, due to the difficulty to exactly detect the influence of the solar signals on the Earth’s climate system. This work applied the Liang–Kleeman information flow method to reveal the causal influence of TSI on OHCA in upper 700 m.

The distribution of the information flow from TSI to OHCA is not homogenous in space. It is because that the dynamical processes of atmosphere and oceans transport and redistribute the incoming heat from solar irradiation. Multi-scale information flow method reveals that the variation of TSI is a cause of the mean OHCA change in upper ocean, especially on the timescale of 10-to-20 years. In contrast, wavelet coherence gives only correlation between them on timescale less than ten years. Since the solar activity contributes to the Earth’s climate change mainly on decadal timescale, the information flow from TSI to OHCA could be overwhelmed by strong dynamic events on inter-annual timescales, for instance, super El Niño. In both Atlantic and Indian Ocean, the information flow on decadal timescale is significant at a 90% confidence level. On inter-annual timescales, it varies due to the strong inter-annual signals.

We also investigated the information flow from TSI to EOFs of the global OHCA. For OHCA mode of ENSO scale, the information flow from TSI is significant in 1970s, when the oscillation of the mode is rather small. The TSI also contributes to the modes of global warming and decadal oscillation. For the global warming mode, the information flow is detected only on timescales of less than 10 years. For the decadal oscillation mode, the information flow is also found on decadal scales, especially before 1980s.

## Methods

### Liang–Kleeman information flow

For time series *X*_1_ and *X*_2_, the information flow *T*_2→1_ from *X*_2_ to *X*_1_ is given by^22–24^

2 $${T}_{2\to 1}=\frac{{C}_{11}{C}_{12}{C}_{2,d1}-{C}_{12}^{2}{C}_{1,d1}}{{C}_{11}^{2}{C}_{22}-{C}_{11}{C}_{12}^{2}},$$

where *C * =  (*C*_*ij*_) is the covariance matrix of *X*_*i*_ and *X*_*j*_, *C*_*i,dj*_ is the covariance matrix of *X*_*i*_ and ${\dot{X}}_{j,n}$, ${\dot{X}}_{j,n}$=$({X}_{j,n+1}-{X}_{j,n})/\Delta t$ is the Euler forward difference sequence of *X*_*j*_.

Zero *T* means no causality. For the causal inference purpose, we only need to consider the absolute value of an information flow. Switching the indices 1 and 2 in Eq. (2) we will get the information flow *T*_1→2_ from *X*_1_ to *X*_2_. The method is also applicable for measuring the causality from a time series to a spatiotemporal field^38^.

Dynamical states are evolutionary; therefore, the information flow should be varying in time. Liang adopted sliding window approach to describe the information flow in different period of time^39^. As they pointed out that the information flow depends on the data length, i.e. the window size, whose choice, however, is empirical. Therefore, the information flow should be interpreted on various timescales, instead of a fixed one. This work adapts the information flow method into a multi-scale version which can measures the information flow for various timescales (window sizes).

First, for a given small window size we use the sliding window approach to calculate information flow. We suggest that the window include at least 30 sampling points, since less data increases the uncertainty of Pearson correlation used in the formula of information flow (Eq. (2)). For each pair of time series, the confidence interval of the information flow is determined via one-tailed test utilized the Monte Carlo simulation (bootstrap). Then, we increase the window size step by step, and in each step, we slide the window and calculate information flow in each time period. Larger window size means fewer sliding of window. Therefore, the illustration of multi-scale information flow is a triangle contour diagram.

### Empirical orthogonal function

Principal component analysis (PCA) compresses the information in a two dimensional matrix in a few dominant patterns. In geophysics, PCA is usually referred as empirical orthogonal function (EOF) analysis. EOF method projects spatial and temporal variability on an orthogonal basis, which is derived by computing the eigenvectors of a spatially weighted anomaly covariance matrix. The eigenvectors, EOFs, present mutually orthogonal spatial patterns of variability. And the expansion coefficients of EOFs are principle components (PCs), showing how the spatial modes oscillate in time.

To make the resulting patterns of classical EOF method more physically interpretable, rotated approach is sometimes performed (for example, by using the varimax rotation method) after getting EOFs. The reconstructed EOFs by rotated EOF method are not necessarily orthogonal.

### Wavelet coherence analysis

Wavelet analysis method decomposes time series into wavelet functions. Each of them is localized in both frequency and time. The continuous wavelet transform is defined as the convolution of a time series, say ${x}_{n}, n=1,\dots ,N$, with the scaled and normalized wavelet. It gives a series of wavelets, say ${W}_{n}^{X}\left(s\right)$, of a time series ${x}_{n}$ with uniform time steps $\delta t$. Here, *s* is the varying timescale on which the wavelet stretched. For continuous wavelet transform, the wavelets should not be orthogonal.

This work applies wavelet coherence to test proposed linkages between two time series. It is defined as^38^

3 $${R}_{n}^{2}(\text{s})=\frac{{\left|S\left({s}^{-1}{W}_{n}^{XY}(s)\right)\right|}^{2}}{S\left({s}^{-1}{\left|{W}_{n}^{X}\left(s\right)\right|}^{2}\right)\cdot S\left({s}^{-1}{\left|{W}_{n}^{Y}\left(s\right)\right|}^{2}\right)},$$

where *S* is a smoothing operator. The cross transform of two time series ${x}_{n}$ and ${y}_{n}$ is defined as ${W}^{XY}={W}^{X}{W}^{Y*}$, where “*” denotes complex conjugation. As that of a traditional correlation coefficient, wavelet coherence can be regard as a localized correlation coefficient in time frequency space, but does not necessarily have high power. A consistent or slowly varying phase lag should be expected if the two series are physically related. The consistent or phase lag can usually be tested against mechanistic models of the physical process. The significance level of the wavelet coherence is also determined using the Monte Carlo simulations.

## Supplementary information

Supplementary information accompanies this paper at 10.1038/s41598-020-74331-2.

Supplementary Information.

## Data Availability

All the data used in this work are available freely in the internet. The TSI data used in this study is available online at Lasp.colorado.edu/lisird/data/nrl2_tsi_P1M/. OHCA data is available in https://www.nodc.noaa.gov/OC5/3M_HEAT_CONTENT.
