# Author Correction: The causality from solar irradiation to ocean heat content detected via multi-scale Liang–Kleeman information flow

**DOI:** 10.1038/s41598-021-86723-z

**Published:** 2021-03-24

**Authors:** Gang Wang, Chang Zhao, Min Zhang, Yuanling Zhang, Min Lin, Fangli Qiao

**Affiliations:** 1 The First Institute of Oceanography, State Oceanic Administration, Ministry of Natural Resources, 6 Xian-Xia-Ling Road, Qingdao, 266061 China; 2 Laboratory for Regional Oceanography and Numerical Modeling, Pilot National Laboratory for Marine Science and Technology, Qingdao, 266237 China; 3 Key Laboratory of Marine Science and Numerical Modeling (MASNUM), Ministry of Natural Resources, Qingdao, 266061 China; 4 Center for Ocean Big Data Research and Applications, National Engineering Laboratory for Integrated Aero-Space-Ground-Ocean Big Data Application Technology, The First Institute of Oceanography, MNR, Qingdao, 266061 China; 5 School of Mathematical Sciences, Ocean University of China, Qingdao, 266071 China

Correction to: *Scientific Reports* https://doi.org/10.1038/s41598-020-74331-2, published online 13 October 2020

The original version of this Article contained errors.

The description of the causality analysis performed in the study was not sufficiently detailed. This is included in the new Supplementary Information file that now accompanies the Article.

Additionally, due to an incorrect path setting in the code, an incorrect dataset was used in the analyses: solar data obtained from^1^ was used instead of the data specified in the Data availability section (which is the correct dataset). All analyses were re-done using the correct source data.

As a result, in the Abstract,

“The results indicate that TSI is a part cause of the El Niño-Southern Oscillation (ENSO) and global warming, especially in the 1970s.”

now reads:

“The results indicate that TSI is a part cause of the El Niño-Southern Oscillation (ENSO), especially in the 1970s.”

In the Results and discussion, subsection ‘Causality from TSI to the global OHCA’,

“During this period the information flow from TSI to OHCA is 0.068. Bootstrap test gives a confidence level of 57%. It is to say that the causality from TIS to the global mean OHCA is not significant on multi-decadal timescale. Sliding window approach (Fig. 1b) with the window size of 22 years shows that in most of 22-year period, the causality is significant at a 68% confidence level estimated by the Monte Carlo test (for variables of a normal distribution, a 68% confidence interval covers variables from one standard deviation below the mean value to one standard deviation above the mean value).”

now reads:

During this period the information flow from TSI to OHCA is 0.0051. Bootstrap test gives a confidence level of 52%. It is to say that the causality from TIS to the global mean OHCA is not significant on multi-decadal timescale. Sliding window approach (Fig. 1b) with the window size of 22 years shows that in most of 22-year period, the causality is not significant even at a low confidence level of 68% estimated by the Monte Carlo test (for variables of a normal distribution, a 68% confidence interval covers variables from one standard deviation below the mean value to one standard deviation above the mean value).”

and

“The information flow is significant (at a 68% confidence level) at the following areas: the mid-and-low latitude of the Atlantic, the South Indian Ocean, the western tropical Pacific, and the east of Australia.”

now reads:

“The information flow is significant (at a 68% confidence level) at the following areas: the South Indian Ocean, the western tropical Pacific, and the east of Australia.”

Furthermore, in the same section,

“Significant correlation is found only in periods shorter than 2 years. The multi-scale information flow (Fig. 3b), however, indicates that the TSI is still a cause of OHCA on longer timescales from decadal to 30 years. On these scales, the information flow from TSI to OHCA is significant at a 90% confidence level. On timescale longer than 40 years, no significant (at a 90% confidence level) information flow from TSI to OHCA is found. Around the two super El Niño events (1982–1983 and 1997–1998), the information flow from TSI to OHCA is small and not significant (at a 90% confidence level). The strong inter-annual oscillation signal of El Niño-Southern Oscillation (ENSO) overwhelms the rather slight decadal signals. After the super El Niño events, the information flow becomes apparent and significant (at a 90% confidence level).”

now reads:

“Significant correlation is found only in periods shorter than 3 years. The multi-scale information flow (Fig. 3b), however, indicates that the TSI is still a cause of OHCA on longer timescales from decadal to 25 years. On these scales, the information flow from TSI to OHCA is significant at a 90% confidence level. On timescale longer than 25 years, no significant (at a 90% confidence level) information flow from TSI to OHCA is found. Around the two super El Niño events (1982–1983 and 1997–1998), the information flow from TSI to OHCA is small and not significant (at a 90% confidence level). The strong inter-annual oscillation signal of El Niño-Southern Oscillation (ENSO) overwhelms the rather slight decadal signals.”

Additionally, in the Results and discussion, in subsection ‘Information flow from TSI to OHCA in the ocean basins’,

“The information flow method, however, reveals significant (at a 90% confidence level) causality from TSI to OHCA on 20–40-year timescales (Fig. 4c). It is consistent with that from TSI to the global OHCA (Fig. 3b). On the other hand, the information flows from TSI to the respective North and South Atlantic represent quite different features on timescales. For the North Atlantic, the information flow is significant on timescales less than 30 years (Fig. 4d); For the South Atlantic, however, it is significant even on timescale of 40 years (Fig. 4e).”

now reads:

“The information flow method, however, reveals significant (at a 90% confidence level) causality from TSI to OHCA on 10–25-year timescales (Fig. 4c). It is consistent with that from TSI to the global OHCA (Fig. 3b). On the other hand, the information flows from TSI to the respective North and South Atlantic represent quite different features on timescales. For the North Atlantic, the information flow is significant on timescales less than 20 years (Fig. 4d); For the South Atlantic, however, it is significant even on timescale of 40 years around 2000 (Fig. 4e).”

and

“On timescale longer than 40 years, there is significant (at a 90% confidence level) information flow from TSI to OHCA in the Indian Ocean and to the South Indian Ocean or the North Indian Ocean (Fig. 5c,d).”

now reads:

“On timescale longer than 40 years, for timeseries whose centers are in 1970–1990, there is significant (at a 90% confidence level) information flow from TSI to OHCA in the North Indian Ocean (Fig. 5c).”

Furthermore, in the Results and discussion, in subsection ‘Timescale of the information flow’,

“Wavelet coherence analysis shows that TSI correlates with each of the PCs (Fig. 7a–c) on inter-annual-to-inter-decadal timescales.”

now reads:

“Wavelet coherence analysis shows that TSI correlates mainly with PC1 (Fig. 7a) on inter-annual timescales, and nearly no significant correlation to PC2 and PC3 (Fig. 7b,c).”

and

“The causality from TSI to PC1 is not significant any more at the 0.1 level (one tailed test).”

now reads:

“On timescales greater than 10 years, the causality from TSI to PC1 is not significant any more at the 0.1 level (one tailed test).”

Finally, in the Conclusions,

“Multi-scale information flow method reveals that the variation of TSI is a cause of the mean OHCA change in upper ocean, especially on the timescale of 20-to-40 years.”

now reads:

“Multi-scale information flow method reveals that the variation of TSI is a cause of the mean OHCA change in upper ocean, especially on the timescale of 10-to-20 years.”

and

“For the global warming mode, the information flow is detected only on timescales of less than 20 years. For the decadal oscillation mode, the information flow is also found at decal scales, especially before 1980s.”

now reads:

“For the global warming mode, the information flow is detected only on timescales of less than 10 years. For the decadal oscillation mode, the information flow is also found on decadal scales, especially before 1980s.”

In addition, changes were made to Figures 1-5 and Figure 7. The figures in the Article were replaced and the original versions are included below as Figures 1, 2, 3, 4, 5 and 6.

**Figure 1:**
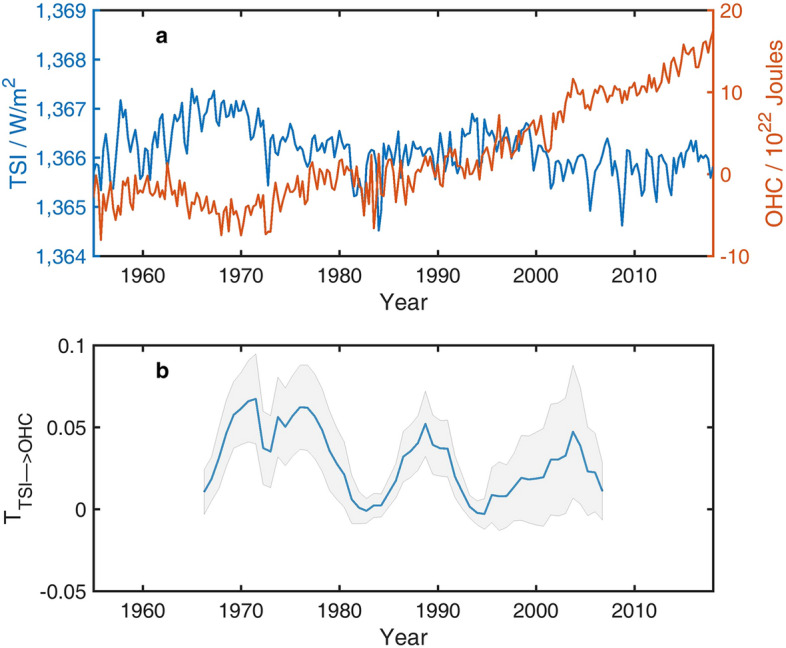
The original incorrect version of Figure 1.

**Figure 2:**
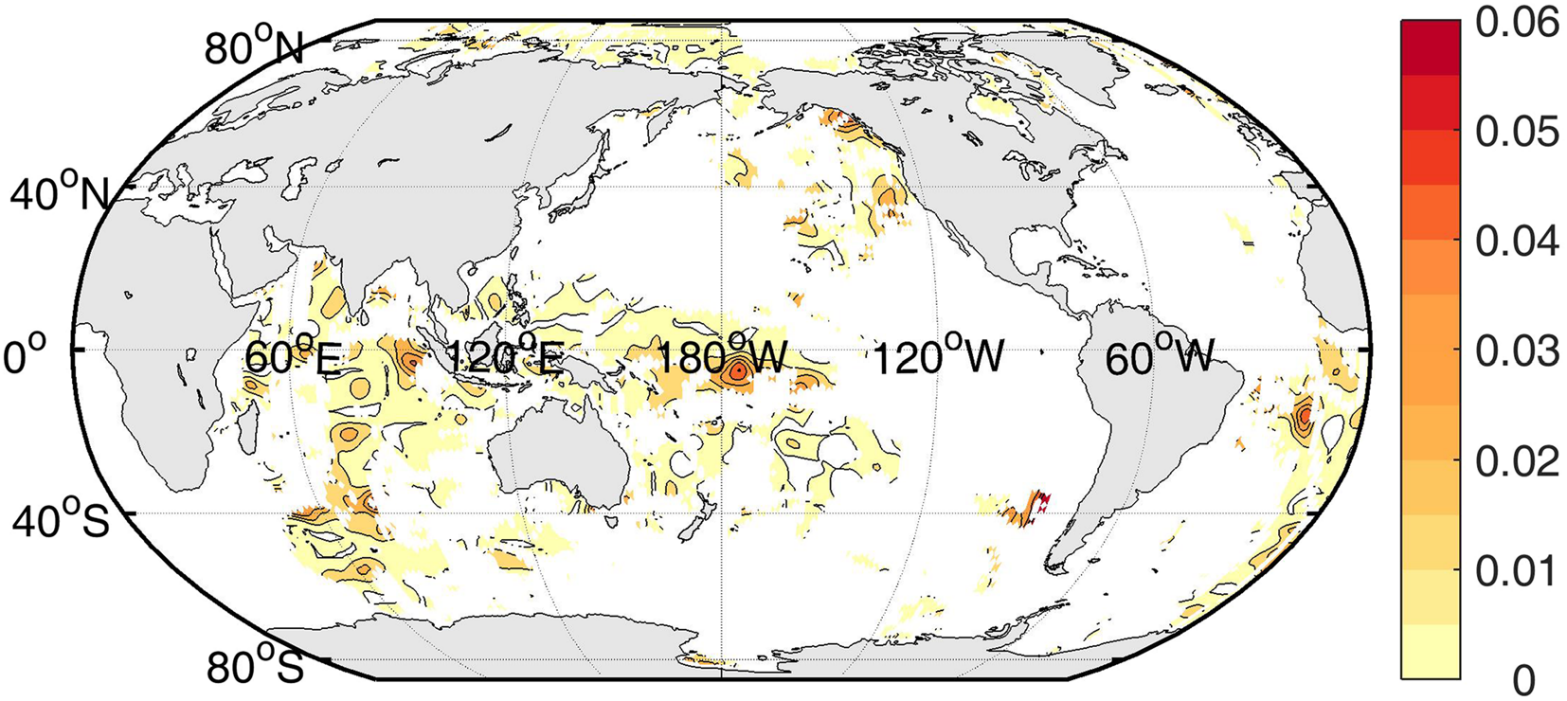
The original incorrect version of Figure 2.

**Figure 3:**
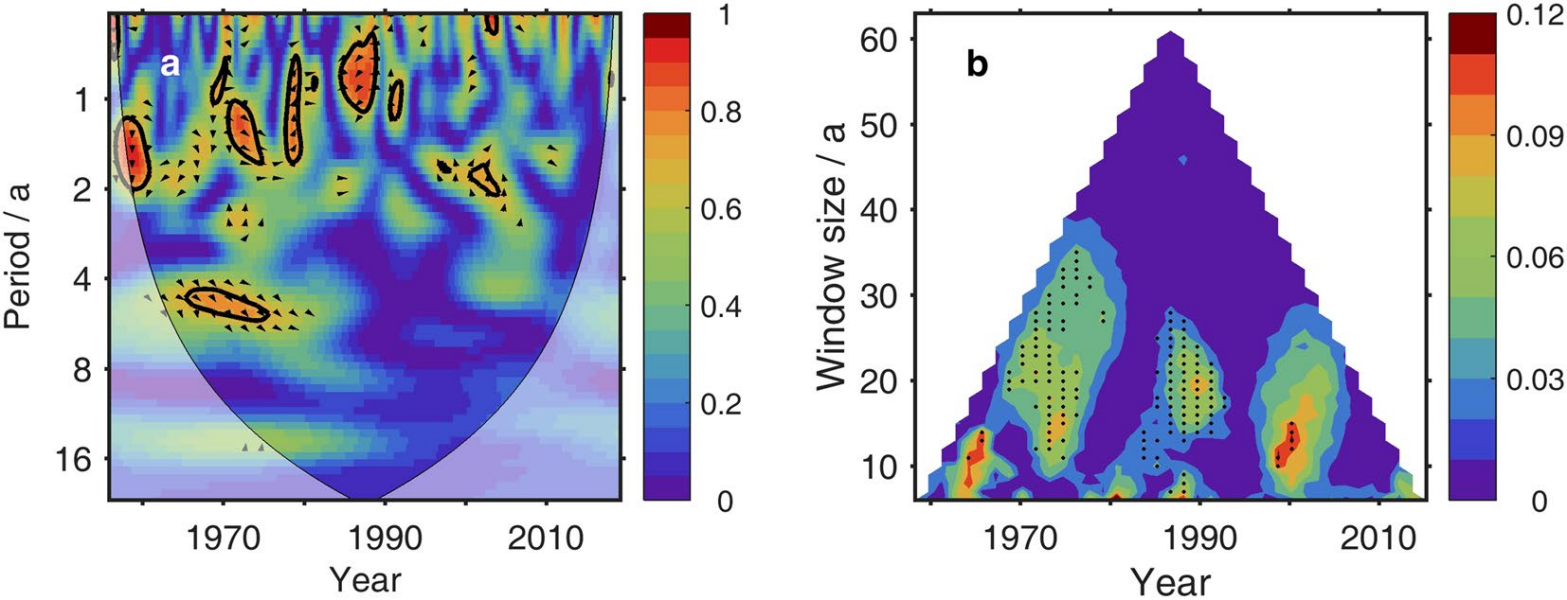
The original incorrect version of Figure 3.

**Figure 4:**
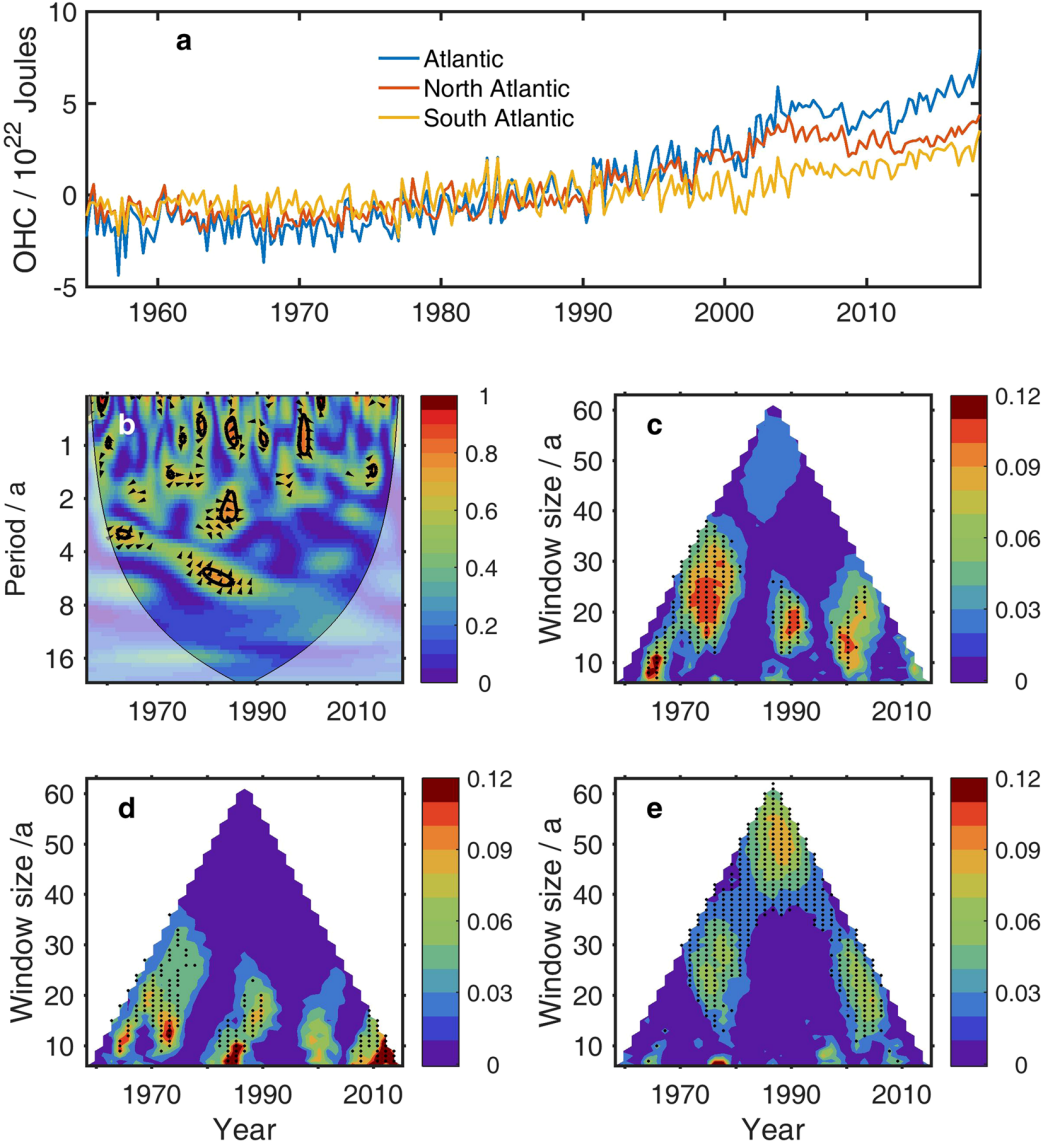
The original incorrect version of Figure 4.

**Figure 5:**
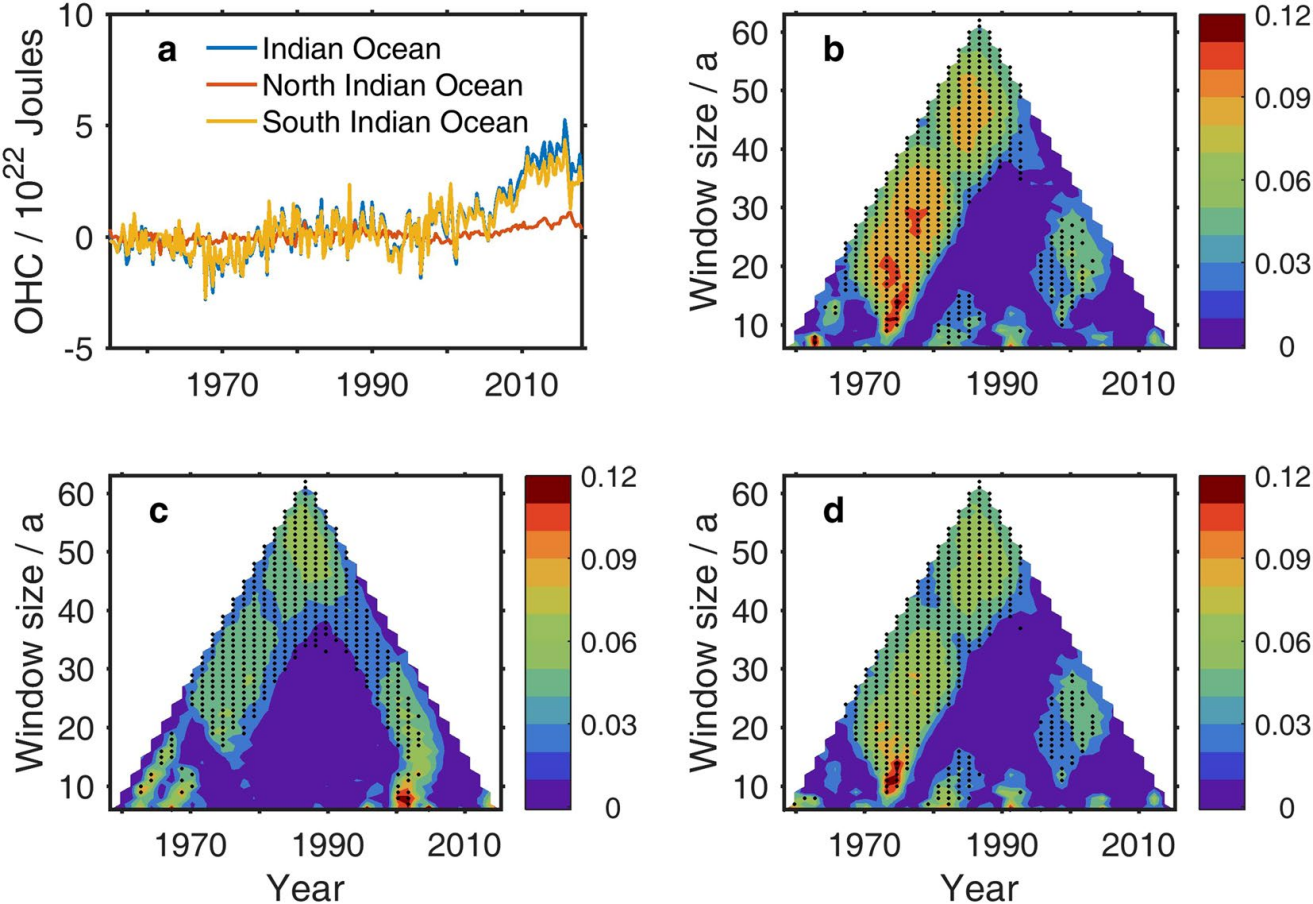
The original incorrect version of Figure 5.

**Figure 6:**
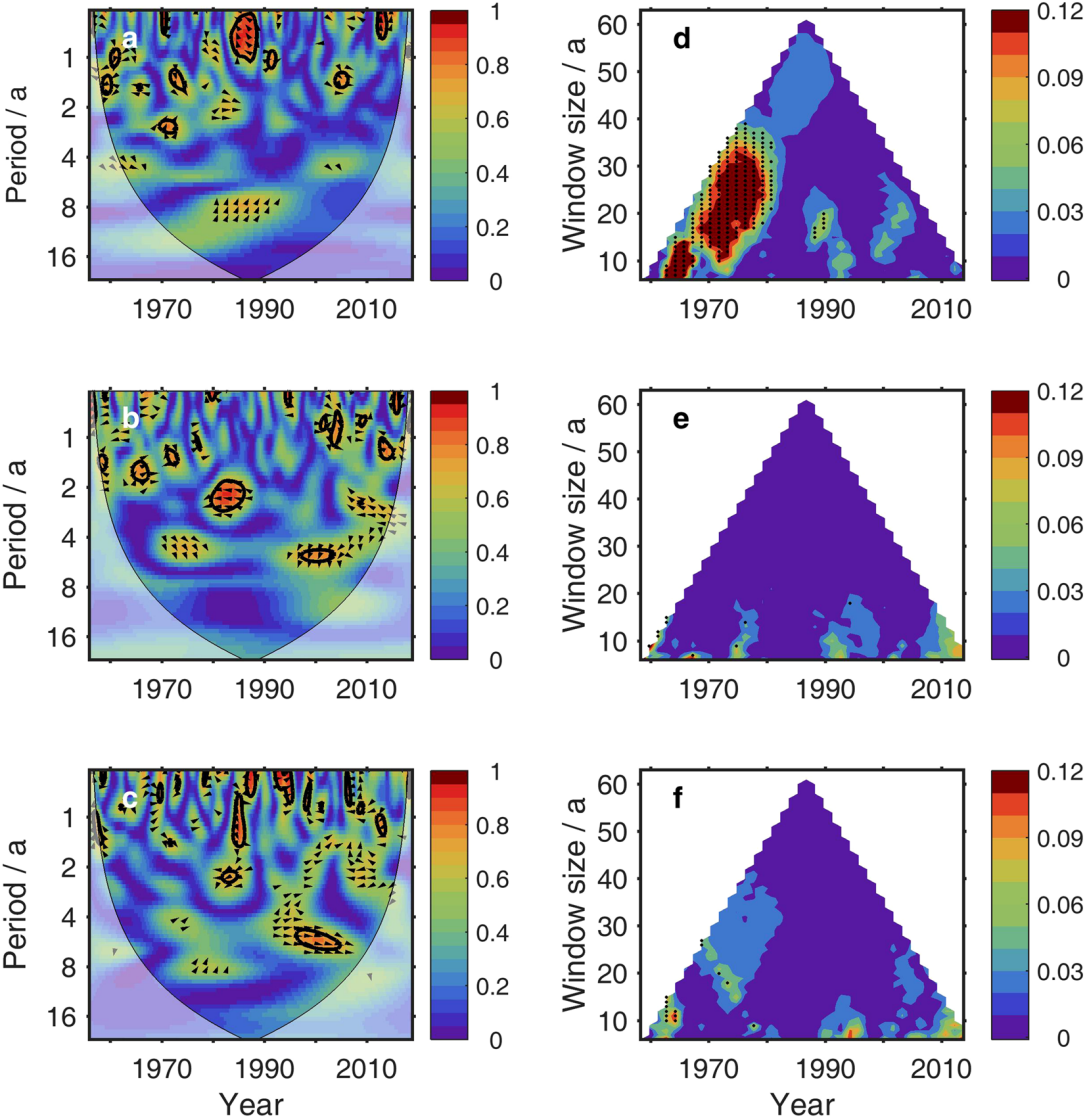
The original incorrect version of Figure 7.

The changes do not affect the main conclusions of the Article. All errors have now been corrected in the PDF and HTML versions of the Article, and in the Supplementary Information file that accompanies the Article.
