# Supplementary material for: The causality from solar irradiation to ocean heat content detected via multi-scale Liang–Kleeman information flow
Source: Sci Rep. 2020 Oct 13;10:17141. doi: 10.1038/s41598-020-74331-2 (PMC7553940; doi:10.1038/s41598-020-74331-2)
Supplement: Supplementary file 1 — Supplementary Information. [file 41598_2020_74331_MOESM1_ESM.docx]

**Supplementary information: The causality from solar irradiation to ocean heat content detected via multi-scale Liang-Kleeman information flow**

Gang Wang*, Chang Zhao, Min Zhang, Yuanling Zhang, Min Lin & Fangli Qiao

Supplementary information to: *Scientific Reports* <https://doi.org/10.1038/s41598-020-74331-2,> published online 13 October 2020.

Here we give a further description on the technique of Liang-Kleeman information flow (LKIF), and its application to the 2D system of solar activity and ocean heat content anomaly.

1. **Liang-Kleeman information flow.** Transfer entropy, the most popular measure for information flow or information transfer, provides a way of measuring causality. There are many extensions of transfer entropy that have been applied to causal coupling estimation from time series. Since information flow or causality is a physical notion, it should be rigorously formulated instead of empirically proposed. Schreiber^1^ suggested a transfer entropy from the viewpoint of Markov processes, which was readily reformulated from known equations of dynamics and was able to distinguish driving and responding elements in systems. Such "rigorous ab initio" exposition and analysis of that transfer entropy was presented by Smirnov^2^, to measure the “dynamical causal effects” for stochastic dynamical systems. Liang and Kleeman^3^ also derived an approach for the measure of causality, Liang-Kleeman information flow, from first principles. It was originally introduced for a system whose dynamics are fully known. The system could be *n*-dimensional, autonomous or non-autonomous. A two-dimensional (2D) case looks like:

$\begin{matrix} \frac{dx_{1}}{dt}=F_{1}(x_{1},x_{2},t) \\ \frac{dx_{2}}{dt}=F_{2}(x_{1},x_{2},t) \end{matrix}$ , (S1)

where $x_{1},x_{2}$ are two state variables those may be either deterministic or random.

Define a joint probability density function on the sample space of $x_{1},x_{2}$, and the entropy function of the joint probability density function in the Shannon sense can be derived. Denote the time evolution of the marginal entropies of $x_{1}$ and $x_{2}$ as $H_{1}$ and $H_{2}$, respectively. The evolution of either of the marginal entropy, say, $H_{1}$ is then replenished from two different sources: one is from $x_{1}$ itself (internal mechanism), and the other from$x_{2}$ (external influence) through the information flow from $x_{2}$ to $x_{1}$. Write the former as $\frac{dH_{1}^{*}}{dt}$ and the latter as $T_{2\to1}$, then

$\frac{dH_{1}}{dt}=\frac{dH_{1}^{*}}{dt}+T_{2\to1}$.

Note that the information flow $T_{2\to1}$is separated out from the inter wined mechanism. After a series of manipulation, Liang and Kleeman derived the expression of $T_{2\to1}$. In the same spirit, Liang^4^ also formulated the information transfer for discrete system. That is, the formula applies to both continuous flows and discrete maps.

Liang^5^ made a further progress for two time series$X_{1}$ and $X_{2}$. He chose a linear model to test the information flow corresponding to a 2D dynamics system. Through maximum likelihood estimator, the rate of information flow from $X_{2}$ to $X_{1}$ is estimated as

$T_{2\to1}=\frac{C_{11}C_{12}C_{2,d1}-C_{12}^{2}C_{1,d1}}{C_{11}^{2}C_{22}-C_{11}C_{12}^{2}}$, (S2)

where *C*=(*C_ij_*) is the covariance matrix of *X_i_* and *X_j_*, *C_i,dj_* is the covariance matrix of *X_i_* and $\dot{X}_{j,n}$, $\dot{X}_{j,n}$=$(X_{j,n+1}-X_{j,n})/\Delta t$ is the Euler forward difference sequence of *X_j_* . This concise formula (Eq. (S2)) bridges the theory and real applications. And it has been put to application to some real world problems^5-9^.

1. **The applicability of Liang-Kleeman information flow.** The formula of LKIF in Eq. (S2) is for a 2D linear system, and has been validated with several kinds of causal inference problems^7^. It was also validated for some nonlinear problems which shows good approximations^4^. For example, the result is obtained for a 2D stochastic system like:

$\begin{matrix} dX_{1}=(a_{1}X_{1}+b_{1}X_{2})dt+c_{1}W_{1}, \\ dX_{2}=a_{2}X_{2}dt+c_{2}W_{2}. \end{matrix}$ (S3)

Clearly, $X_{2}$ drives $X_{1}$, but not vice versa.

In fact, the basic idea of LKIM was first illustrated with a nonlinear system with randomness limited to its initial condition (Eq. (S1)). The measure has been applied to nonlinear 2D systems. For example, Stips et al.^8^ applied the LKIF in revealing the causal structure between CO_2_ and global temperature, a nonlinear system in the real world.

The derivation of LKIF shows that it is a causality measure in the sense of Shannon entropy, which is quite different from correlative analysis methods. LKIF measure possesses a property of asymmetry between the involved parties, while correlation analysis put the two events on an equal stance.

1. **Significant test of information flow.** For two given time series A and B, the measure of LKIF from A to B could not tell us to what extent A influences B. The statistical significance test of the results should be performed. In the example of total solar irradiance (TSI) and ocean heat content anomaly (OHCA), the data length is limited and their distribution is unknown, especially for the multi-scale version when the window size is rather small. Given a significance level, we estimate the confidence interval for Eq. (S3) with bootstrap.

Bootstrap is a computer-intensive resampling method for estimating the sampling distribution when information about the data distribution is absent. The method aims to determine the probability distribution from the data itself, without recourse to central limit theorem. It is a kind of non-parametric statistical method which tries to estimate the error but not to reduce it. Monte Carlo approximation is used for the bootstrap estimate. For a pair of given data, create artificial indices of the values by randomly drawing from the data sets to constitute the corresponding bootstrap sample, and calculate the LKIF between the two data sets. Each element in the data sets could be picked more than once. In this work, this procedure is independently repeated 1000 times to yield the distribution of the information flows. For a given confidence level, we can give the percentile confidence interval of the LKIF. This goal is obtained by using the toolbox in Matlab (bootstrp.m).

A detailed introduction to the bootstrap please refers to Hesterberg^10^.

Anyhow, we don’t think that any LKIF passed the significant test by bootstrap is meaningful. If the significant LKIF measures are sparsely distributed as that given in our multi-scale contour maps, it should not be treated as a meaningful result.

1. **Multi-scale Liang-Kleeman information flow.** The multi-scale Liang-Kleeman information flow is given as follows:

For a fixed small window size, we use sliding window approach to calculate the LKIF between the data in the window. For each pair of time series, the confidence interval corresponding to the information flow is determined via one-tailed test utilized the Monte Carlo simulation (bootstrap). Then we increase the window size and calculate the LKIF on a longer timescale, until the window size equals the whole length of data. Larger window size means fewer windows could be chosen. Therefore, the presentation of the multi-scale LKIF is a triangle, as was given in the following figures (for instance, Fig. S3).

1. **Estimates of LKIF in two directions between TSI and OHCA.** As the principle of nil causality states: an event is not causal to another event if the evolution of the latter does not depend on the former. However, it does not mean that causality from one of the directions (from A to B, or from B to A) should be zero. If fact, there are causal interactions observed in real-world phenomena. For example, the mutual causation system being composed of the Didinium and Paramecium^11^.

The system of TSI and OHCA is a good example to validate the LKIF method, since its dynamics is quite clear. We can expect that TSI is a causal to OHCA, but OHCA has no feedback to the TSI. The information flow therefore should be one directional (from TSI to OHCA). In fact, although the system may have different formula from the dynamical system described in Eq. (S3), it is very similar to the latter: TSI is coupled in the entropy variation of OHCA, while OHCA does not involves in the variation of TSI.

Here we compare the estimates of LKIF in two opposite directions – from TSI to OHCA ($T_{2\to1}$) and from OHCA to TSI ($T_{1\to2}$). Physically, those estimates of $T_{1\to2}$ should be zero. Since the noise is inevitable, the ‘zero’ should be in the sense of statistical significance. The LKIFs given in the supplementary figures are from OHCA to TSI, that is, $T_{1\to2}$.

Figure S1 gives the information flow from the global mean OHCA to TSI. $T_{1\to2}$ is comparable with $T_{2\to1}$(Fig. 1b) but quite different from the latter. It’s not surprising. Neither direction gives significant LKIF for a sliding approach with a given window size. It’s the limitation of sliding window approach of LKIF, and also the reason why we adapted the LKIF method into a multi-scale version. Anyhow, the results reveal asymmetry of the LKIF, which is quite different from correlative analysis methods.

In Fig. 2, $T_{2\to1}$ is significant (at a 68% confidence level) at some hot spot regions: the South Indian Ocean, the western tropical Pacific, and the east of Australia (Fig. 2). It gives much more meaningful information than $T_{1\to2}$ (as shown in Fig. S2) does. In fact, $T_{1\to2}$ defines nearly no meaningful area where OHCA is a possible causal to TSI. It accords with our knowledge that the OHCA should not be a causal to TSI.

In Fig. 3b, multi-scale $T_{2\to1}$ reveals significant (at a 90% confidence level) causality from TSI to OHCA on decadal or longer timescales. The corresponding $T_{1\to2}$ (Fig. S3), however, gives a sharp contrast. Only several sparsely distributed points in the contour map are significant at a 90% confidence level. We could say, those points do not make any sense. They appear by chance either in time domain or in timescale domain, and should not be seemed as meaningful signals.

The same discussion applies to rest of the figures (Figs. S4-S5). In neither Atlantic (Figs. S4a-c) nor Indian Ocean (Figs. S4d-f), there is meaningful information from OHCA to TSI by the multi-scale LKIF, indicating that OHCA is not a causal to TSI. So does for the principle components of the global OHCA (Fig. S5).

Although the formula of Eq. (S2) seems applicable for any pair of time series, we suggest it be applied to linear system or nonlinear system with randomness limited to its initial condition. We should also bear in mind that the two series should physically coherent. To yield a meaningful result, the physical dynamics beneath the data should be known in advance. It is also a basic requirement of correlative analysis methods. For two time series having nothing to do with each other, high correlation in the Pearson sense may be found.

In the system of TSI and OHCA, we have already known that TSI does have impact on OHCA, but not vice versa. What we expect to find by using LKIF measure is the details of causality in different periods and different time scales. If we are not sure about it, any result from the LKIF measure is meaningless. Meaningful results should be explained physically.

## Supplementary Figures


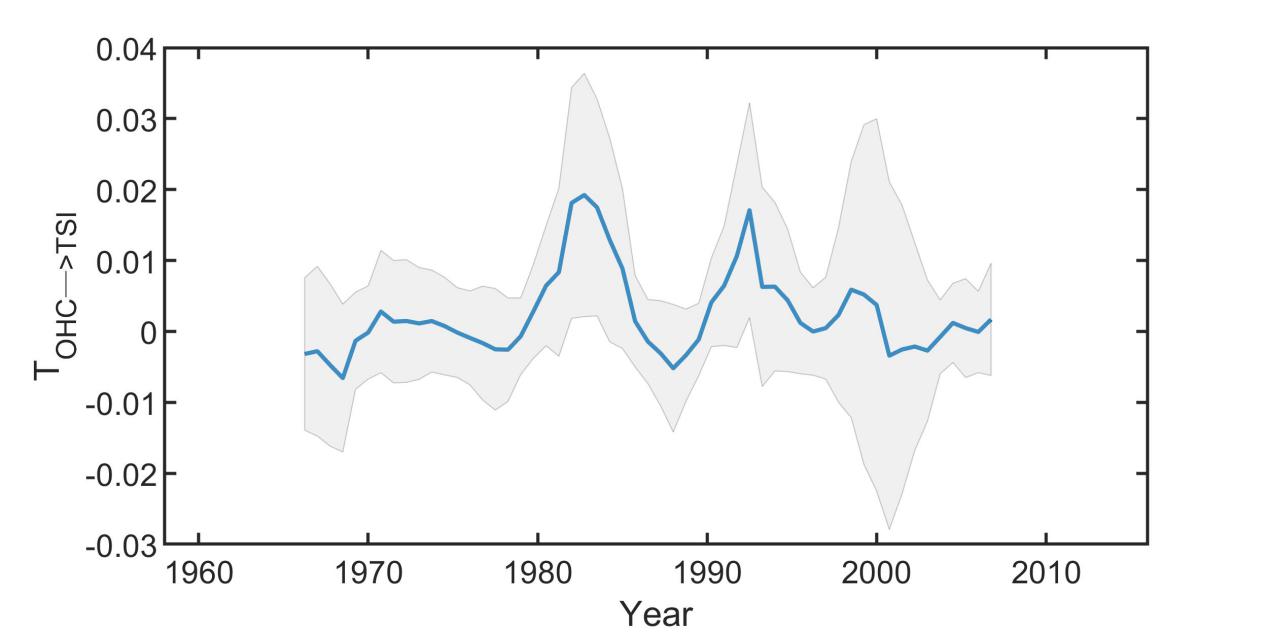


**Figure S1**. Information flow from the global mean OHCA to TSI. The size of the sliding window is 22 years (88 points), and the gray shadow covers a 68% confidence interval. The figure was created by using Matlab version R2016b.

**Figure S2.** Global information flow from the gridded global OHCA in upper 700 m to TSI during the period of 1955 to 2017. Grids at which the confidence level is less than 68% are shaded blank. The map was created by using the m-map toolbox (https://www.eoas.ubc.ca/~rich/map.html) for Matlab (version R2016b).


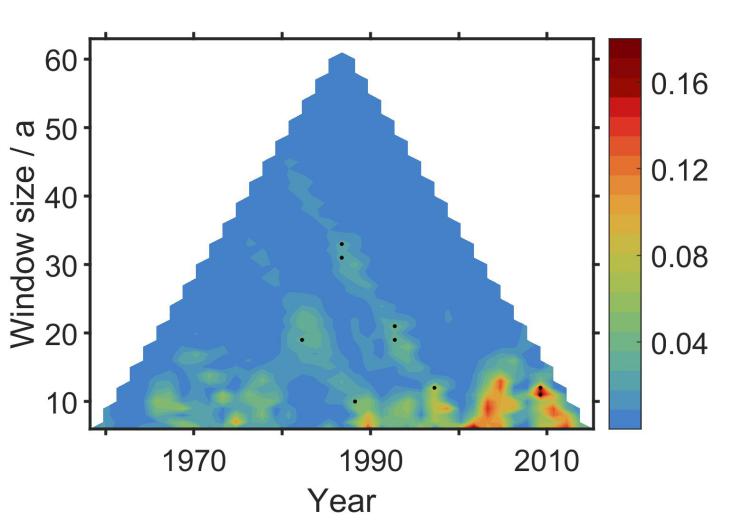


**Figure S3**. Multi-scale information flow from the global OHCA time series to TSI. It reveals no causality from OHCA to TSI on decadal or longer timescales. The black dots denote that the information flow is significant at a 90% confidence level. The figure was created by using Matlab version R2016b.


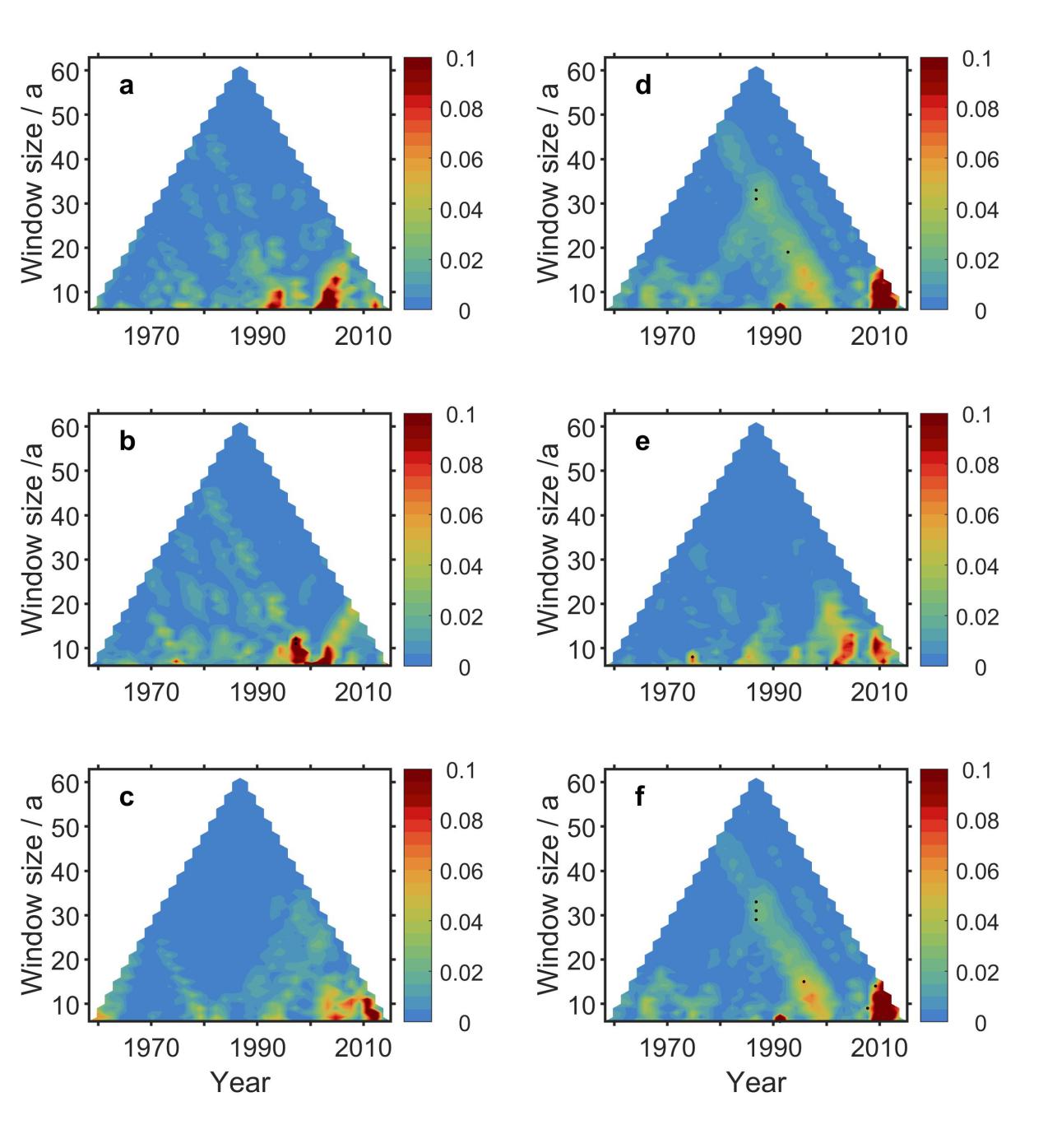


**Figure S4**. Left column: Multi-scale information flow from OHCA in the Atlantic (**a)**, North Atlantic (**b**) and South Atlantic (**c**) to TSI, respectively; Right column:　Multi-scale information flow from OHCA in the Indian Ocean (**d**), North Indian Ocean (**e**), and South Indian Ocean (**f**) to TSI, respectively. The black dots denote that the information flow is significant at a 90% confidence level. The figures were created by using Matlab version R2016b.


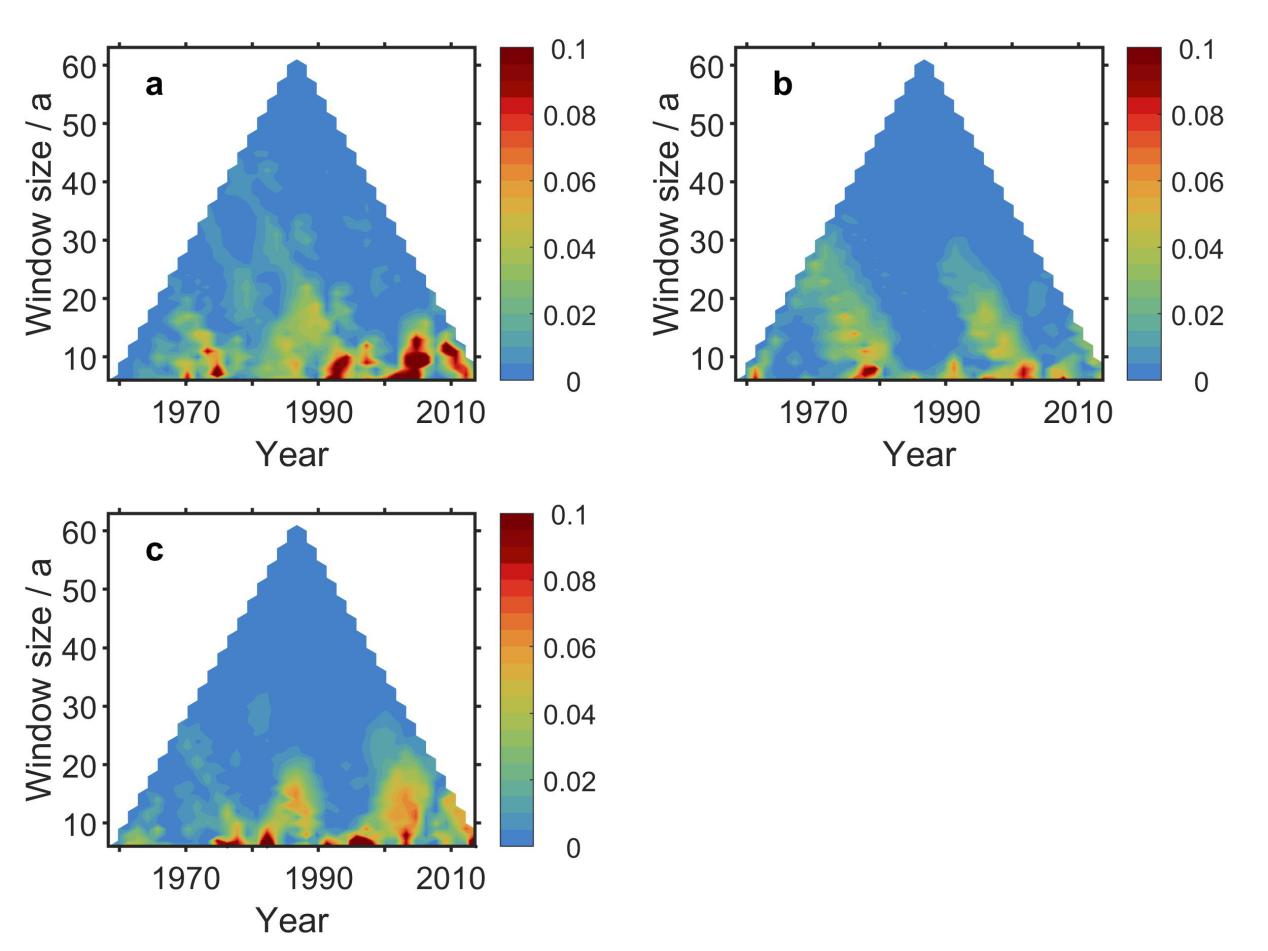


**Figure S5.** Multi-scale information flow from PC1 (**a**), PC2 (**b**) and PC3 (**c**) of the global OHCA to TSI, respectively. The black dots denote that the information flow is significant at a 90% confidence level. The figures were created by using Matlab version R2016b.

## References

1. Schreiber, T. Measuring information transfer. *Phys. Rev. Lett.* **85**, 461–464 (2000).
2. [Smirnov](https://www.researchgate.net/scientific-contributions/Dmitry-A-Smirnov-2187055260?_sg%5b0%5d=A3ayJWuLw1_WVZZGMr-iWq9E-rXvWSBGJHBv6wT2zM9UM9r5yKKunSdcyLwd1hVvZJ0tPTY.6RJjSrGZSS7AZua0fukR9w8LZB2TwRyAMw_vwoqgdId8CM-2IDA27RcwuAM76udmtusjnRsDDhaZ_OeeKwhhJQ&_sg%5b1%5d=PXS_6df-X0YeuPMtsuZWcNZ4if-CWuZAssJeRrbo8a_kviIajV6tjRzd241kPnayQfrMH6A.CXXGtKzlkwiFPXZ7aCKDOaM7rWj58pQ5o7KYqRFatA3095CuAfAub3o3bUvNjJvZToKa0N2_Rawpj-q0XUGW4w), D. A. Transfer entropies within dynamical effects framework. *Phys. Rev. E* **102**(6), doi: [10.1103/PhysRevE.102.062139](https://www.researchgate.net/deref/http://dx.doi.org/10.1103/PhysRevE.102.062139?_sg%5b0%5d=59QciJF1OrFHG9IapltWQ_QmXB0RBcq8Iz0KSj6UHw9Oif4WEpITWdVTlsqtjBUcF_jYJodQvg3heU7GmEhtV7BQgg.ESg9Obt2Og5qKgCJ2-s9AuFNYJQCav6K-sTqTGMnBPVwUAogUxdq6nvueFM_2JqlUAw-hoa2r0o36xBEtjOrhA) (2020).
3. Liang, X. S. & Kleeman, R. Information transfer between dynamical system components. *Phys. Rev. Lett.* **95**, 244101 (2005).
4. Liang, X. S. The Liang-Kleeman information flow: theory and applications. *Entropy* **15**, 327–360 (2013).
5. Liang, X. S. Unraveling the cause-effect relation between time series. *Phys. Rev. E* **90**, 052150 (2014).
6. Liang, X. S. Exploring the big data using a rigorous and quantitative causality analysis. *J. Comput. Commun.* **4**, 54–60 (2016a).
7. Liang, X. S. Information flow and causality as rigorous notions ab inito. *Phys. Rev. E* **94**, 052201-1-28 (2016b).
8. Stips, A., Macias, D., Coughlan, C., Garcai-Gorriz, E. & Liang, X. On the causal structure between CO_2_ and global temperature. *Sci. Rep.* **6**, 21691 (2016).
9. Bai, C., Zhang, R., Bao, S., Liang, X. & Guo, W. Forecasting the tropical cyclone genesis over the Northwest Pacific through identifying the causal factors in the cyclone-climate interactions. *J. Atmos. Ocean Tech.* **35**, 247–259 (2018).
10. Hesterberg, T. Bootstrap. *Wires Comput. Stat.* **6**, 497–526 (2011).
11. Yang, A. C., Peng, C-K. & Huang, N. E. Causal decomposition in the mutual causation system. *Nat. Commun.* **9**, 3378 (2018).
